# Unearthing Grape Heritage: Morphological Relationships between Late Bronze–Iron Age Grape Pips and Modern Cultivars

**DOI:** 10.3390/plants13131836

**Published:** 2024-07-03

**Authors:** Mariano Ucchesu, Anna Depalmas, Marco Sarigu, Massimo Gardiman, Andrea Lallai, Franco Meggio, Alessandro Usai, Gianluigi Bacchetta

**Affiliations:** 1 Institut des Sciences de l’Evolution de Montpellier (ISEM), University of Montpellier-CNRS-IRD-EPHE, 34000 Montpellier, France; 2 Dipartimento di Scienze Umanistiche e Sociali (DUMAS), Università di Sassari, 07100 Sassari, Italy; depalmas@uniss.it; 3 Centro Conservazione Biodiversità (CCB), Dipartimento di Scienze della Vita e dell’Ambiente (DISVA), Università degli Studi di Cagliari, Viale Sant’Ignazio da Laconi, 13, 09123 Cagliari, Italy; andrea.lallai@unica.it (A.L.); bacchet@unica.it (G.B.); 4 Council for Agricultural Research and Economics, Research Centre for Viticulture and Enology (CREA-VE), 31015 Conegliano, Italy; massimo.gardiman@crea.gov.it; 5 Department of Agronomy, Food, Natural Resources, Animals and Environment (DAFNAE), University of Padova, 35020 Padova, Italy; franco.meggio@unipd.it; 6 Interdepartmental Research Centre for Viticulture and Enology (CIRVE), University of Padova, Via XXVIII Aprile 14, 31015 Treviso, Italy; 7 Soprintendenza Archeologia, Belle Arti e Paesaggio per la Città Metropolitana di Cagliari e le Province di Oristano e Sud Sardegna, 09123 Cagliari, Italy; alessandro.usai@cultura.gov.it

**Keywords:** ancient cultivars, domestic grape, morphometrics, viticulture, *Vitis*

## Abstract

The grapevine was one of the earliest domesticated fruit crops and has been cultivated since ancient times. It is considered one of the most important fruit crops worldwide for wine and table grape production. The current grape varieties are the outcome of prolonged selection initiated during the domestication process of their wild relative. Recent genetic studies have shed light on the origins of the modern domestic grapevine in western Europe, suggesting that its origin stems from the introgression between eastern domestic grapes and western wild grapes. However, the origin of ancient grapevines remains largely unexplored. In this study, we conducted an extensive analysis of 2228 well-preserved waterlogged archaeological grape pips from two sites in Sardinia (Italy), dated to the Late Bronze Age (ca. 1300–1100 BC) and the Iron Age (4th and 3rd centuries BC). Using morphometrics and linear discriminant analyses, we compared the archaeological grape pips with modern reference collections to differentiate between wild and domestic grape types and to investigate similarities with 330 modern cultivars. Grape pips from the Late Bronze Age displayed a high percentage of similarity with domesticated grapevines, with a small percentage assigned to wild ones, while the majority of grape pips from the Iron Age were classified as domestic. Discriminant analyses revealed that both white and red grape varieties were cultivated during the Late Bronze and Iron Ages, suggesting a high level of diversification in grape cultivation. Furthermore, a high percentage of archaeological grape pips from both periods showed strong similarities with modern cultivars from the Caucasus and Balkans. This suggests that the great diversity of grapevines present in Sardinia could result from interbreeding between western Asian cultivars and local grapevines that began in the Late Bronze Age. Additionally, a substantial proportion of archaeological grape pips exhibited similar morphometric characteristics to two important Mediterranean grape cultivars: “Muscat à petits grains blancs” and “Garnacha”.

## 1. Introduction

Within the genus *Vitis* L., the domestic grapevine [*Vitis vinifera* subsp. *sativa* (DC.) Hegi] is considered one of the most important fruit crops worldwide for wine and table grape production [1,2]. Morphological and genetic studies revealed that the current grape cultivars are the outcome of prolonged selection that began during the domestication process of its wild relative *Vitis vinifera* L. subsp. *sylvestris* (W illd.) Hegi [3,4,5]. Archaeobotanical and genetic analyses indicate that the first domestication took place in western Asia [6,7,8]. Recent genetic studies conducted on 3525 cultivated and wild accessions by Dong et al. [9] unveiled that two distinct domestication events occurred concurrently approximately 11,000 years ago in western Asia and the Caucasus region. These two domestication events are believed to have occurred due to the climatic changes during the last glaciation, which contributed to the diversification and geographic distribution of the wild progenitor throughout Eurasia, generating two distinct wild populations in the Near East and the Southern Caucasus. From these two wild progenitors, two separate domestication processes of the grapevine emerged [9].

Domestication induced phenotypic changes in wild relatives, such as variations in seed shape, where domestic grapes typically exhibit elongated seeds with a long beak, while wild grapes have smaller, roundish seeds with a short beak [10]. Archaeobotanical evidence from the 4th millennium BC onwards reveals an increasing presence of grape remains in the northern Levant and Near East, suggesting a correlation with the emergence of complex societies and the onset of grapevine cultivation, and thanks to the intensification of trade exchanges, potentially contributing to the spread of viticulture [3].

Similarly, in Italian Bronze Age sites, large concentrations of grape pips have been discovered, indicating the possibility of the adoption of viticulture during this period [11,12,13,14]. Different genomic analyses have suggested the possibility of parallel or secondary grape domestication events within regions surrounding the Mediterranean Basin [15,16]. However, the hypothesis of a second grape domestication event occurring outside the original primary domestication centre remains a subject of debate [17]. The secondary domestication hypothesis suggests that different communities, possibly across various regions of the Mediterranean Basin, where the wild progenitor of the grape is abundantly present, independently recognized the value of local wild grape varieties. These communities might have consciously or unconsciously selected and cultivated these varieties, starting domestication processes [11,16]. This concept aligns with the broader idea of convergent evolution in agriculture, where distinct societies independently developed similar agricultural practices and domesticated similar plants in different geographic regions. 

The second hypothesis suggests that the origin of domesticated grapes in western Europe is the result of introgression events, a process facilitated by the transfer of genes through repeated backcrossing, which can occur naturally through pollen transfer by wind or insects or be assisted by human intervention in agricultural practices [17].

Recent genetic analyses conducted on modern domesticated and wild grapes suggested that the origin of domesticated grapes in western Europe was the result of introgression between domesticated grapes from the Near East and the ancient wild populations present in western Europe [9].

The morphological analysis of archaeological grape pips can offer crucial insights into their classification as wild or cultivated, shedding light on the onset of grape domestication. This technique utilises elliptic Fourier transforms (EFTs) to convert an object’s outline into shape descriptors known as Fourier coefficients (EFDs). The EFDs are then applied as geometric morphometric features in multivariate analyses to identify various morphological types [18].

In different studies, morphometric analysis, conducted through the use of the elliptic Fourier transform (EFT) method, and applied to archaeological grape pips, has demonstrated the validity of distinguishing wild grapes from domestic ones [11,18,19,20,21,22,23].

Archaeological grape pips preserved in waterlogged conditions are excellent samples for morphological comparisons with modern materials as they lack the typical morphological deformations observed in charred and mineralised seeds [23].

Recent morphometric studies conducted on waterlogged grape pips from the archaeological site of Sa Osa (CW Sardinia, Italy), dated to the Late Bronze Age (ca. 1300–1100 BC), showed the presence of morphologically domestic grape pips [11,14,22]. Similarly, a study combining morphometric and paleogenomic analyses revealed the presence of domestic grape pips dated to the Middle Bronze Age (ca. 1450–1200 BC) in southern Italy [13]. The domestic grape pips found in Sardinia and southern Italy currently represent the oldest evidence of early grape cultivation in the western Mediterranean [11,13]. At present, we do not know whether these domestic grapes resulted from a secondary domestication process or an ancient introgression event between domestic grapes introduced from outside and local wild grapes. 

In this light, we employed morphometric and discriminant analysis to investigate the domestication status of a new, large dataset of well-preserved waterlogged grape pips dated to the Late Bronze Age (ca. 1300–1100 BC) and Iron Age (4th–3rd centuries BC). The aims also extend to identifying possible morphological relationships with modern grape cultivars. The objectives of this work are to deepen our understanding of the origins of grape domestication in Italy and contribute to exploring the diversity of cultivated grapevines in ancient times in the western Mediterranean.

## 2. Results

### 2.1. Comparison of Archaeological Pips with Modern Reference Materials: Wild/Domestic Morphotypes

Morphometrical analysis of grape pips is a well-established method for distinguishing between wild and domestic varieties, based on consistent differences in seed shape and size that emerged during domestication.

**Figure 1:**
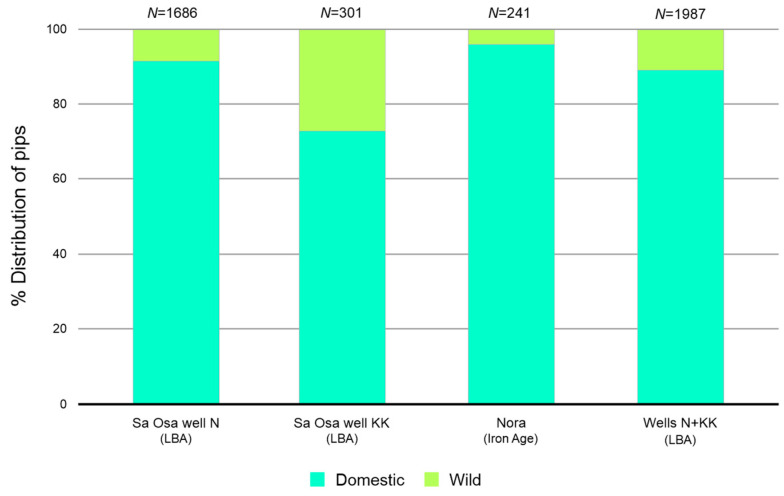
Identification of the wild/domestic status of the archaeological pips. Total number of archaeological pips (*N*), Late Bronze Age period (LBA).

Morphometrical data of archaeological grape pips (*N* = 2228) from two waterlogged archaeological sites were subjected to discriminant analysis, which compared them with modern wild and domestic grape varieties. Linear Discriminant Analysis (LDA) showed an overall correct classification of 95.7%. LDA carried out on the pips (*N* = 1686) from the Late Bronze Age site of Sa Osa (well N) revealed that 92% (*N* = 1559) were classified as domestic and 8% (*N* = 127) as wild (Figure 1). Meanwhile, the grape pips (*N* = 301) from well KK showed that 74% (*N* = 223) were classified as domestic and 26% (*N* = 78) as wild (Figure 1). From the Iron Age site of Nora, grape pips (*N* = 241) revealed that 97% (*N* = 235) were classified as domestic and 3% (*N* = 6) as wild (Figure 1).

The high percentage of domestic grape pips at these sites strongly suggests established viticulture practices in Sardinia during the Late Bronze Age and Iron Age periods.

### 2.2. Comparison of Archaeological Pips with Individual Modern Grape Cultivars

Based on the previous LDA results, we conducted a second LDA using only the archaeological grape pips that showed domestic morphology, and we compared them with individual modern cultivars. In this analysis, we compared the archaeological pips, which were included as unknown samples, with 145 white, 183 red and 3 pink modern grape cultivars. Comparing archaeological pips to modern cultivars helps us understand the diversity of ancient grape varieties and potentially trace the origins and spread of specific cultivars.

**Figure 2:**
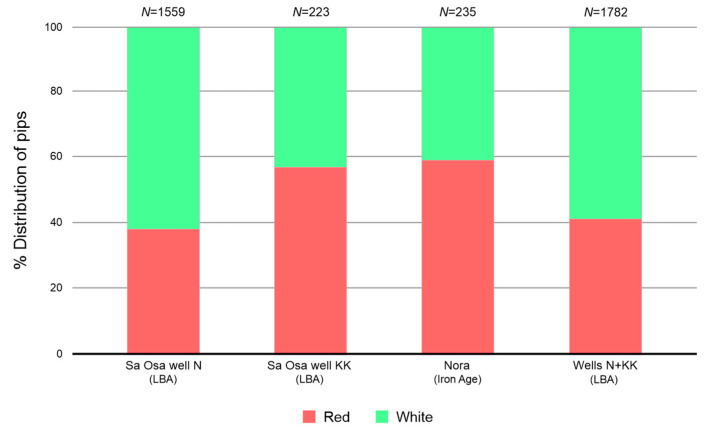
Percentage distribution of archaeological pips from each site, according to the grape skin colour of identified cultivars. Total number of archaeological pips (*N*), Late Bronze Age period (LBA).

From the Late Bronze Age site of Sa Osa (well N), the archaeological grape pips were similar to 65 cultivars, with 29 red (38%) and 36 white cultivars (62%), respectively (Figure 2). Specifically, among the red cultivars, most of the archaeological pips were assigned to “Forzarin” (*N* = 88), “Lambrusco viadanese” (*N* = 86), “Gregu nieddu” (*N* = 83) and “Garnacha tinta” (*N* = 81), (Table 1 and Table S1). Among the white cultivars, most of the archaeological grape pips were assigned to “Muscat à petits grains blancs” (*N* = 232), “Vitouska” (*N* = 154), “Bayan shirei” (*N* = 123) and “Bourboulenc” (*N* = 121) (Table 1 and Table S1). From well KK, the archaeological grape pips were similar to 62 cultivars, with 30 red (57%) and 32 white cultivars (43%), respectively (Figure 2). Specifically, among the red cultivars, most of the archaeological pips were assigned to “Garnacha tinta” (*N* = 29) and “Gregu nieddu” (*N* = 19) (Table 1 and Table S1). Among the white cultivars, most of the archaeological grape pips were assigned to “Malvasia Dubrovacka” (*N* = 15) and “Muscat à petits grains blancs” (*N* = 11) (Table 1 and Table S1).

From the Iron Age site of Nora, the archaeological grape pips were assigned to 49 cultivars, with 24 red (59%) and 25 white cultivars (41%), respectively (Figure 2). Specifically, among the red cultivars, most of the grape pips were assigned to “Garnacha tinta” (*N* = 51) (Table 1 and Table S1). Among the white cultivars, most of the archaeological grape pips were assigned to “Garnacha blanca” (*N* = 21) (Table 1 and Table S1). The presence of similarities to both red and white grape cultivars in the archaeological samples indicates a diverse grape cultivation landscape in ancient Sardinia, possibly reflecting different uses for wine production and table consumption.

### 2.3. Geographical Origin Analysis of Archaeological Grape Pips

**Figure 3:**
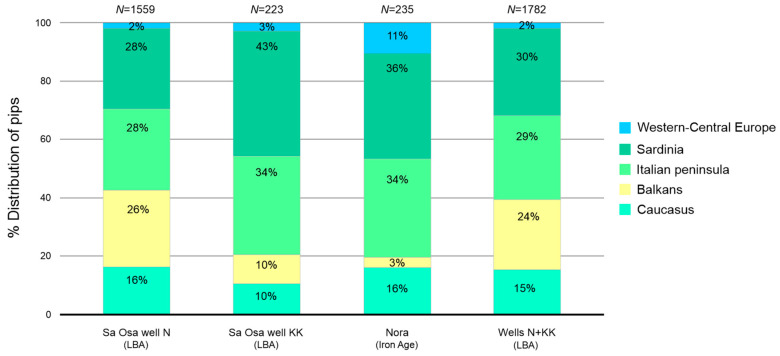
Percentage distribution of archaeological pips from each site, according to the geographical origin of identified cultivars. Total number of archaeological pips (*N*), Late Bronze Age period (LBA).

Analysing the percentage allocation of archaeological pips and considering the geographical origin of individual modern cultivars, the grape pips from the Late Bronze Age of Sa Osa (well N) showed greater similarity with the cultivars from the Italian peninsula and Sardinia, with a percentage of allocation of 28% (Figure 3). Other pips were assigned to the Balkans (26%) and Caucasus (16%), and a small percentage were assigned to central western Europe (2%) (Figure 3). In contrast, the archaeological pips from well KK showed a greater similarity with the cultivars from Sardinia (43%) and the Italian peninsula (34%) (Figure 3). Other pips were equally assigned to the Balkans and the Caucasus (10%), and a small percentage were assigned to central western Europe (3%) (Figure 3).

The pips from the Iron Age of Nora showed greater similarity with the cultivars from Sardinia (36%) and the Italian peninsula (34%), while the remaining grape pips were assigned to the cultivars from the Caucasus (16%) and central western Europe (11%) (Figure 3). This geographical distribution analysis suggests complex patterns of grape cultivation and potential trade or cultural connections across the Mediterranean region during these periods. The high similarity to Sardinian and Italian peninsula cultivars may indicate local domestication or adaptation, while similarities to Balkan and Caucasus varieties could reflect ancient trade routes or earlier waves of grape introduction.

## 3. Discussion

### 3.1. Domestication and Cultivation of Grapes: Evidence from Late Bronze and Iron Ages Sardinia

In this study, for the first time, we undertook a thorough characterisation of a large number of well-preserved waterlogged grape pips from two archaeological sites in Sardinia, spanning from the Late Bronze Age (ca. 1300–1100 BC) to the Iron Age (4th–3rd centuries BC). By applying morphometrics and LDA, we compared the archaeological grape pips with modern reference collections to differentiate between wild and domestic morphotypes and investigate ancestral forms of current cultivars. It is important to note that while morphometric similarities suggest relationships between ancient and modern cultivars, they do not definitively prove direct lineage. Genetic studies would be necessary to confirm these connections. Our approach allows us to trace potential ancestral relationships between ancient and modern grape varieties, providing insights into the development of viticulture in the region.

Our analyses revealed that most of grape pips from the Late Bronze Age were classified as domesticated, with a small percentage classified as wild, while most of grape pips from the Iron Age were classified exclusively as domesticated. This study confirms the results obtained in another work, where morphometric analyses demonstrated the beginning of grape selection and cultivation practices in Sardinia during the Late Bronze Age and the advancement of grape cultivation in the Iron Age in Italy [14].

Additionally, the archaeological grape pips from both periods showed similarities with different modern grapes, including both white and red cultivars. For the Late Bronze Age, our analyses demonstrated the presence of a high percentage of white grapes compared to red grapes, while during the Iron Age, red grapes were predominant.

The presence of white grapes identified in the Late Bronze Age site suggests that this phenotypic characteristic, exclusively belonging to domestic grapes, was present in the early stages of grape cultivation in Sardinia. The transition from red-berried wild relatives to domestic white grapes marks a pivotal moment in the history of viticulture domestication [9]. 

In grapes, the berry skin colour is regulated by the transcription factor family *VvMybA* [24,25]. White berry skin colour is considered one of the selective traits associated with domestication syndrome [9]. Additionally, genetic analyses have revealed that the presence of heterozygous SNP states in *V. vinifera* subsp. *sylvestris* indicates the existence of white berry alleles within natural wild populations before grapevine domestication [9]. 

However, despite the presence of these alleles in wild grape populations, most wild fruits growing in their natural habitat typically exhibit dark skin colours—primarily red, black, or blue. This phenotypic characteristic appears to result from birds selectively dispersing only dark-skinned fleshy fruits [26]. Several studies have demonstrated that fruit selection by birds is influenced by the nutritional content, which can be indicated by the dark colour of the fruits. The accumulation of anthocyanin signals fruit ripening [26,27,28,29]. Therefore, fruits with dark skin seem to be more successful at being dispersed in the natural environment than lighter fruits [30,31]. For this reason, white grapes hardly appear in wild populations. Therefore, the identification of white grape cultivars in archaeological samples could represent strong evidence of grape domestication. Further evidence that might indicate the use of domestic grapes could be provided by chemical analyses of ceramic residues when wine is detected. Different chemical studies conducted on organic residues from archaeological ceramics, based on the absence of syringic acid (a marker of red wine), suggest that the wine contained in these vessels was made from white grapes [32,33,34,35,36,37,38]. This chemical evidence, in combination with the study of grape pip morphology, could help determine whether the grapes used for wine production were domestic, as wine can potentially also be produced from wild grapes. Indeed, our analysis shows similarities between the archaeological grape pips and modern white cultivars from the Caucasus and the Balkans, indicating a possibility of an influx of domestic cultivars from these regions. However, it is crucial to interpret these results cautiously, as morphological similarities do not necessarily imply direct genetic relationships or continuity of cultivation.

The cultivation of white grape cultivars, in addition to being part of the diet for direct consumption, might also be linked to wine production. Recent chemical analyses conducted on the organic residues of some ceramics dated to the Late Bronze Age in Sardinia [37] and from Pilastri di Bondeno in the Po Valley [34] have highlighted the presence of white wine, supporting the hypothesis that the wine produced was made using domestic white grapes.

### 3.2. Tracing the Ancestry of Modern Grapes: Archaeological Pips Reveal Morphometric Relationships to Modern Varieties

Regarding the identification of similarity with modern cultivars, our morphometric analyses linked the archaeological grape pips from both periods to modern cultivars from the Caucasus, the Balkans, the Italian peninsula and central western Europe. Although these similarities do not allow for a definitive identification of specific cultivars—since our database does not include all existing modern cultivars and may lack those that have become extinct—they do offer insights into the morphological relationships between archaeological grape pips and modern cultivars.

The archaeological grape pips from the Late Bronze Age displayed a high percentage of similarities to white cultivars: “Muscat à petits grains blancs” (Greece/Italy/France), “Bayan shirei” (Armenia and Azerbaijan), “Vitovska” (Slovenia), “Bourboulenc” (France) and “Trebbiano abruzzese” (Italy). Genetic studies have established that “Muscat à petits grains blancs” has been the main founder of the Muscat family, contributing to the great diversity of the Italian germplasm [39,40]. Historical sources first mention Muscat cultivars in Italy in 1300 AD, attesting to their ancient cultivation in the Italian viticultural landscape [41]. Moreover, the ancient origin of Muscat cultivars has been suggested by recent genetic analyses, which hypothesize the emergence of the Muscat flavour around 10,500 years ago [9]. Similarly, our study has highlighted the presence of grapes sharing morphometric characteristics with “Muscat”, suggesting the existence of ancestral grapes related to the Muscat family during the second millennium BC. Future paleogenetic studies on these archaeological samples could provide more definitive evidence of the presence of Muscat-related varieties in ancient Sardinia.

With reference to “Bayan shirei”, some authors attribute its origin to the regions of Armenia and Azerbaijan, where it currently represents an important grape cultivar for wine production [42,43]. Another cultivar that shares morphological similarity with the archaeological grape pips is “Vitovska”, a minor white grape cultivar native to the Karst region, which spans northeastern Italy and western Slovenia. Its name may originate from the local Slovenian dialect or from the village of Vitovlje in the Vipavska Dolina region [42]. Recent DNA parentage analysis has revealed that “Vitovska” is an offspring of “Malvasia bianca lunga” and “Glera”, formerly “Prosecco tondo” [44]. Additionally, our analyses have also revealed similarities with “Malvasia bianca lunga”. Currently, we do not know if these cultivars were the possible founders of the traditional Sardinian cultivars, as comprehensive genetic studies of Sardinian minor traditional grape cultivars have not yet been conducted.

The other cultivar that shares morphometric traits with archaeological pips from Sa Osa is “Bourboulenc”, an ancient cultivar from the Vaucluse region in Provence, southern France [42]. It was likely first mentioned as Borbolenques in Cavaillon in 1515, but its introduction to Sardinia remains unknown [41]. Previous morphometric studies highlighted the existence of morphometric relationships between the archaeological grape pips and “Bourboulenc”, e.g., the pips from the Middle Bronze Age (ca. 1450–1200 BC) of Pertosa Cave (southern Italy) [13], the Iron Age (7th century BC) of Samos Heraion (eastern Aegean) [20] and the Iron Age (ca. 1300–1000 BC) of Digomi Room (Tbilisi, Georgia) [21]. This evidence seems to indicate a close relationship between the archaeological grape pips recovered from various archaeological sites and “Bourboulenc”, suggesting an ancient origin for this grape cultivar.

Furthermore, a high percentage of grape pips from Sa Osa displayed similar morphometric characteristics to different red cultivars such as “Forzarin”, “Lambrusco viadanese”, “Gregu nieddu”, “Garnacha tinta” and “Aptiche aga” (Armenia). With reference to the cultivars “Forzarin,” “Lambrusco viadanese,” and “Gregu nieddu,” there is currently no certain information about their origins. The “Forzarin” cultivar is grown in the province of Pordenone in Friuli and northeastern Italy, while the “Lambrusco viadanese” cultivar is mainly cultivated in the provinces of Mantua and Cremona (in Lombardy) [42]. Meanwhile, “Gregu nieddu” is a minor grape cultivar grown in Sardinia. Genetic analyses indicate that it is the result of a cross between “Heben x Monastrell”, two grape cultivars that have produced many traditional Sardinian cultivars. Meanwhile, “Aptiche aga” is a traditional cultivar from Armenia, and no more information is available about its parentage relations with western grape cultivars. However, recent paleogenetic analyses conducted on two pips from well N at the Sa Osa site have shown that they shared the same chorotype present in modern grape cultivars from Armenia [45]. This evidence, together with the results of our analyses, supports the hypothesis that cultivars from the Caucasus were introduced to Sardinia during the Bronze Age. The identification of similarities to both "Garnacha tinta" and "Garnacha blanca" in our samples raises interesting questions about the early diversification of grape varieties.

In reference to the origins of “Garnacha tinta” and “Garnacha blanca”, both identified at the Late Bronze Age and Iron Age sites, we do not have definitive information about their origins [42]. Historical sources first mention Garnacha in Spain in 1513 AD and in Sardinia in 1549 AD, sparking a debate about the cultivar’s place of origin [42,46]. “Garnacha tinta” represents an important grape cultivar cultivated in Sardinia (called “Cannonau”), Spain and France for wine production [42]. Genetic analyses have established that “Garnacha tinta” and “Grenache blanc” share the same genetic profile, differing only in somatic mutations of berry colour [47,48]. Additionally, genetic analyses have shown that “Cannonau”, along with various Sardinian cultivars, is genetically much closer to cultivars from Armenia and Georgia than to others from the Italian peninsula, suggesting a different history of viticulture in Sardinia [49]. These genetic analyses support the results obtained in our study, as archaeological pips from both archaeological sites showed similarity with cultivars from the Caucasus and the Balkans. Moreover, the archaeological grape pips from both periods showed a high degree of similarity with traditional cultivars from Sardinia. This indicates that these traditional cultivars share morphological characteristics with the first ancestral grapevines cultivated by Sardinian communities in the past. 

### 3.3. The Influence of Eastern Mediterranean Cultivars on Ancient Sardinian Viticulture

The introduction of the allochthonous cultivars from the eastern Mediterranean likely occurred due to the numerous contacts established by the Bronze Age communities (circa 1600–930 BC) of Sardinia with the island of Cyprus and subsequently with the Carthaginian colonists during the Iron Age (circa 930–600 BC). Archaeological documentation attests to the discovery of ox-hide-shaped copper ingots from Cyprus at several Sardinian Bronze Age sites, indicating the existence of regular trade contacts between the eastern peoples and the protohistoric communities of Sardinia [50,51]. Most likely, these commercial exchanges enabled the communities of Sardinia to acquire allochthonous grape cultivars from the eastern Mediterranean, as well as knowledge of grape cultivation techniques. 

The results of this study highlighted how archaeological grape pips were morphologically similar to some modern grape cultivars, suggesting that these cultivars may have played a significant role in contributing to the great diversity present in the viticultural landscape of the western Mediterranean and, in particular, in Sardinia. While our study provides valuable insights into ancient grape cultivation in Sardinia, it is important to acknowledge that morphometric analysis alone cannot definitively prove the exact varieties present or their origins. Integrating these findings with paleogenetic and archaeobotanical studies could provide a more comprehensive understanding of ancient viticulture in the region.

## 4. Materials and Methods

### 4.1. Modern Materials

Modern reference materials consisted of 330 cultivars of *V. vinifera* subsp. *sativa* originating from western and central Europe (*N* = 173), the Mediterranean area (*N* = 137) and southwest Asia (*N* = 20). Modern grapevine pips were obtained from the germplasm repository of the Council for Agricultural Research and Economics—Research Centre for Viticulture and Enology (CREA-VE), Conegliano, Italy, Agenzia per la Ricerca Scientifica della Regione Autonoma Sardegna (AGRIS), Julius Kühn Institute (JKI) of Quedlinburg (Germany), National Wine Agency of Georgia, Tbilisi, Georgia and the Centre de Ressources Biologiques de la Vigne, Domaine de Vassal-Montpellier (INRAE). Wild grapes consisted of 22 accessions from France (*N* = 3), Italy (*N* = 11), Spain (*N* = 1), Greece (*N* = 3) and the Caucasus area (*N* = 4) and were obtained from the collection of the ISEM-CNRS University of Montpellier, from the Sardinian Germplasm Bank (BG-SAR) of the University of Cagliari and from the National Wine Agency of Georgia, Tbilisi, Georgia.

### 4.2. Archaeological Sites and Materials

**Figure 4:**
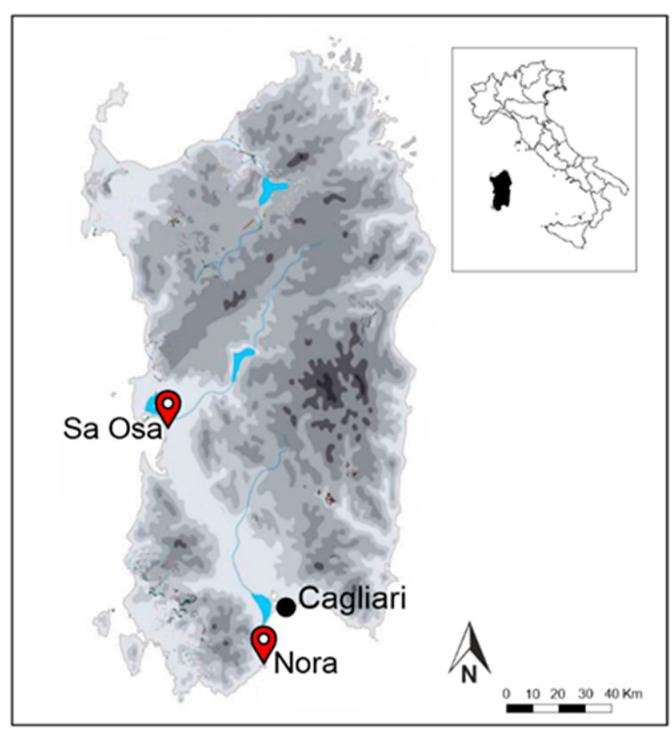
Locations of the archaeological sites of Sa Osa and Nora.

The 2228 archaeological grape pips come from two waterlogged sites located in Sardinia, Italy (Figure 4). The archaeological grape pips used in this study were extracted from the sediment and cleaned of impurities. Subsequently, to avoid any deformation, the materials were kept in deionized water and stored at 5 °C in the Germplasm Bank of Sardinia (BG-SAR) at the Centre for Conservation of Biodiversity at the University of Cagliari.

The first site is Sa Osa, located in central west Sardinia, excavated between 2008 and 2009 by the Soprintendenza Archeologica di Cagliari and the Università di Sassari. These excavations uncovered several wells from the Late Bronze Age (ca. 1300–1100 BC), which yielded thousands of well-preserved grape pips [52]. Previous research using morphometric analyses identified the presence of domestic grapes [11,22]. However, in those studies, the grape pips were compared exclusively to modern grape cultivars from Sardinia, without conducting an extensive comparative analysis with modern individual cultivars originating from the Mediterranean and eastern regions. 

In this study, we utilised a new dataset of archaeological pips from well N (*N* = 1686) and well KK (*N* = 301), totaling 1987 pips.

We compared these with modern grape cultivars from western and central Europe, the Mediterranean Basin, and southwest Asia.

Other waterlogged pips came from the ancient city of Nora (southern Sardinia), where archaeological excavations documented the presence of a Phoenician emporium from the mid-8th century BC onwards, facilitating trade and communication between the eastern Mediterranean and Sardinia [53]. By the end of the 6th century BC, the area came under Carthaginian control. Archaeological excavations conducted between 1978 and 1984 at Nora recovered several transport amphorae from the seabed. From one transport amphora (78 A2), dated to the Iron Age (4th–3rd centuries BC), 663 grape pips were recovered [54]. For this study, we selected only intact grape pips, totaling 241 well-preserved grape pips.

### 4.3. Morphometric Analyses

**Figure 5:**
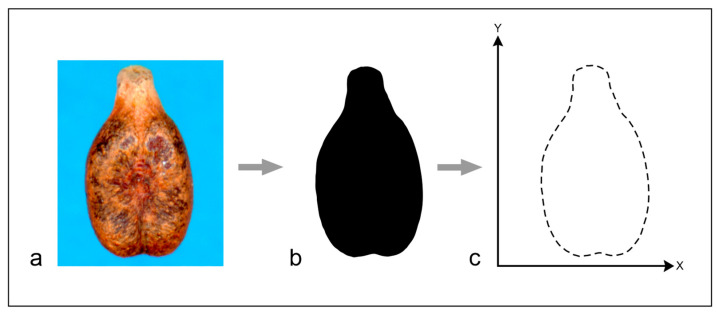
Graphical representation of the different phases of morphometric analyses; (**a**) 600 dpi scanned image, (**b**) black silhouettes of the pip, (**c**) diagram of elliptic Fourier transform (EFT) measurement of 360 reference points.

The dorsal views of digital images of modern and archaeological grape pips were acquired using a flatbed scanner (Epson Perfection V550), with a digital resolution of 600 dpi for a scanning area not exceeding 5100 × 7019 pixels [55]. The grape pip images were converted to black silhouettes using the software package ImageJ v. 1.54 [56] (Figure 5).

The analysis of pip outlines was conducted through the use of the elliptic Fourier transform (EFT) method, according to the methodology described in previous studies [18,57,58]. The EFT method transforms contour geometry into “Fourier coefficients,” sampling outline coordinates (x, y) at 360 evenly spaced points along each outline. Before EFT calculation, the outlines were normalised, considering both the centroid size and the position of the first point relative to the centroid. Following established research protocols [18,30], only the coefficients from the first six harmonics were employed to characterise the view, totaling 24 coefficients (four coefficients per harmonic, one view). This decision, based on six harmonics, strikes a balance between shape description accuracy (encompassing over 95% of total harmonic power) and minimizing measurement errors, which typically rise with harmonic rank [58]. The outline analyses were conducted using ImageJ v. 1.54 and a specific plugin developed by Diaz [59].

### 4.4. Statistical Analysis

Statistical data were executed using IBM SPSS v. 16.0 (SPSS 2006), applying stepwise Linear Discriminant Analysis (LDA). This method is commonly used to classify or identify unknown groups characterised by quantitative and qualitative variables [60]. It allows for finding the combination of predictor variables to minimize the within-class distance and maximize the between-class distance simultaneously, thus achieving maximum class discrimination [61,62]. The stepwise method uses three statistical variables, Tolerance, F-to-enter and F-to-remove, to identify and select the best features that will be used to characterise the seed samples. The Tolerance value indicates the proportion of residual variance not explained by other independent variables in the equation. F-to-enter and F-to-remove values characterise the influence of each variable in the model and provide insight into the effects of adding or removing a variable. At each stage, the variable with the highest F-to-enter value that exceeds the entry criterion (F > 3.84) is incorporated into the model. Variables not included in the final analysis have F-to-enter values below 3.84 and are not added further. The process terminates automatically when no remaining variables can enhance discriminative ability.

Based in previous studies [19,20,58], to ensure the quality of the classification, we utilised a balanced reference material for the LDA in which an identical number (*N* = 1319) of wild and domestic randomly selected pips was present. Finally, a cross-validation procedure was executed to evaluate the performance of the identification system by testing individual unknown cases and classifying them based on all other cases. The validation method employed here was leave-one-out cross-validation (LOOCV). This technique uses one individual case from the original sample as the validation dataset, while the rest of the cases serve as the training set [63,64]. Each case is assigned to a group based on classification functions calculated from all data except the case currently being classified. The leave-one-out estimate of misclassification is determined by the proportion of cases incorrectly classified after sequentially excluding each case’s effect one at a time.

Discrimination among archaeological specimens and modern references was carried out considering the chronological period to which the archaeological remains belong.

## 5. Conclusions

Our study contributed to understanding the complex history of viticulture in Sardinia. By analysing grape pips from two archaeological sites dating back to the Late Bronze Age and the Iron Age, we elucidated the dynamics of grape cultivation and provided valuable insights into the emergence of viticulture over time. During the Late Bronze Age, most of the grape pips were classified as domestic, with a small percentage assigned to the wild category, while the majority of grape pips from the Iron Age were classified as domestic. Evidence of white and red grape cultivation from both periods suggests an advanced stage of viticulture, potentially influenced by intentional introgression with external domestic cultivars. Morphometric analyses linked the archaeological grape pips to modern cultivars from the Caucasus and the Balkans, suggesting that the great diversity of grapevines in Sardinia resulted from interbreeding between eastern cultivars and local grapevines. Additionally, a high percentage of archaeological grape pips exhibited similar morphometric characteristics to two important Mediterranean grape cultivars: “Muscat à petits grains blancs” and “Garnacha”. Although we are still far from fully understanding the intricate history of the origins of modern grapevines, we hope that this research will stimulate new studies that combine paleogenetic and morphometric analyses to build a more detailed picture of viticulture history.

## Figures and Tables

**Table 1:** Number of archaeological grape pips assigned to modern cultivars. In this table, cultivars represented by at least 10 pips are reported (see Table S1 for the complete list). In brackets, the prime names of the grape cultivars reported on the *Vitis* International Variety Catalogue (VIVC) are depicted. The “Use” column in Table 1 indicates whether the modern cultivar is primarily used for wine production (W), table grape consumption (T) or both (W/T), providing insight into the potential uses of similar grapes in antiquity.

Cultivar Name	Origin	Use	Colour	Well N	Well KK	Nora	Total
Moscato bianco (Muscat à petits grains blancs)	Greece/Italy/France	W	white	232	11	2	245
Vitouska	Slovenia/Italy (Friuli)	W	white	154	8	5	167
Cannonanu (Garnacha tinta)	Italy (Sardinia)	W	red	81	29	51	161
Bayan shirei	Azerbaijan	W/T	white	123	3	14	140
Claretta di Sardegna (Bourboulenc)	Italy (Sardinia)	W	white	121	4	0	125
Gregu nieddu	Italy (Sardinia)	W	red	83	19	9	111
Forgiarin (Forzarin)	Italy (Friuli)	W	red	88	2	0	90
Trebbiano abruzzese	Italy (central south)	W	white	83	1	5	89
Lambrusco viadanese	Italy (central)	W	red	86	2	0	88
Aptiche aga	Armenia	W/T	red	66	3	0	69
Malvasia di Casorzo	Italy (north)	W	red	58	3	3	64
Caddiu bianco	Italy (Sardinia)	W	white	51	5	2	58
Grenache blanc (Garnacha blanca)	France	W	white	21	4	21	46
Malvasia bianca lunga	Italy (Tuscany)	W	white	40	0	0	40
Pignolo	Italy (Friuli)	W	red	16	17	4	37
Guleiman kara	Uzbekistan	ND	red	18	3	13	34
Uvalino	Italy (north)	W	red	2	8	19	29
Arvesiniadu	Italy (Sardinia)	W	white	24	4	0	28
Chaouch blanc	Turkey	T	white	13	6	2	21
Schiava	Italy (north)	W	red	13	7	0	20
Malvasia di Sardegna (Malvasia Dubrovacka)	Italy (Sardinia)	W	white	4	15	1	20
Impigno	Italy (south)	W	white	16	1	0	17
Kypreiko	Greece	W	red	16	1	0	17
Tzitzka	Georgia	W/T	white	9	4	4	17
Licronaxu	Italy (Sardinia)	W/T	white	13	1	2	16
Culupuntu	Italy (Sardinia)	W	white	0	0	11	11
Coda di volpe bianca	Italy (Campania)	W/T	white	8	0	2	10
Albourla rose	Ukraine	W/T	red	8	1	1	10
Mazzese	Italy (Tuscany)	W	red	0	5	5	10

## Data Availability

Data are contained within the article and Supplementary Materials.

## Supplementary Materials

The following supporting information can be downloaded at https://www.mdpi.com/article/10.3390/plants13131836/s1, Table S1: Number of archaeological grape pips assigned to modern cultivars.
